# Fabrication of Color Glass with High Light Transmittance by Pearlescent Pigments and Optical Adhesive

**DOI:** 10.3390/ma15072627

**Published:** 2022-04-02

**Authors:** Hyeon-Sik Ahn, Akpeko Gasonoo, Seong-Min Lim, Jae-Hyun Lee, Yoonseuk Choi

**Affiliations:** 1 Department of Electronic Engineering, Hanbat National University, Daejeon 34158, Korea; princass123@naver.com (H.-S.A.); seongmin625@naver.com (S.-M.L.); 2 Department of Chemistry, University of Calgary, 2500 University Drive N.W., Calgary, AB T2N 1N4, Canada; akpeko.gasonoo@ucalgary.ca; 3 Department of Creative Convergence Engineering, Hanbat National University, Daejeon 34158, Korea; jhyunlee@hanbat.ac.kr

**Keywords:** BIPV, pearlescent pigment, NOA, spin coating, high transmittance, lamination process

## Abstract

In this study, we propose a solution process for realizing colored glass for building integrated photovoltaic (BIPV) systems by spin coating a color solution composed of pearlescent pigments mixed in a Norland Optical Adhesive (NOA) matrix. Color solutions are made from mixing pearlescent pigments in NOA63. Compared to a physical vapor deposition process, color coatings are achieved by spin coating in a relatively simple and inexpensive process at room temperature. The optical properties can be easily controlled by adjusting the spin coating speed and the concentration of the pearlescent pigments. The produced colored glass achieved a high transmittance of 85% or more in the visible wavelength range, except for the wavelength spectrum exhibiting the maximum reflectance. In addition, we propose a one-step lamination process of colored glass on a solar cell by leveraging on the adhesive property of the NOA matrix. This eliminates the cost and process of additional ethylene vinyl acetate (EVA) layer or other materials used in the conventional lamination process. The colored glass produced through this study has stability that does not change its properties over time. Therefore, it is expected to be applied to the BIPV solar module market where aesthetics and energy efficiency are required.

## 1. Introduction

Recently, the efficient production and consumption of eco-friendly renewable energy has become an important research subject due to climate change. From an architectural design point of view, research on a system that generates electricity using solar energy and can be integrated into the exterior wall of a building is of high interest and has been actively investigated [1,2,3,4]. A novel approach of building integrated photovoltaic (BIPV) system on the outer wall of the building has been developed, and many architectural structures based on this have been proposed [5,6]. The BIPV system, which comprises solar cells, is mainly installed on the roof of a building or on the outer wall of a building, which has a relatively large area to efficiently produce solar energy in buildings. The building to which the BIPV system is applied is eco-friendly and can be self-sufficient or energy-saving, making it economical and very efficient [7,8]. Through the large-scale application and integration of solar energy technology, it becomes possible to generate energy in every kind of building. A significant portion of the generated electricity is consumed within the built environment, and the power distribution and transportation costs by the smart grid is significantly reduced [8]. To apply the BIPV system, various factors, such as the efficiency of the solar panel, the transmittance of the front colored glass, the insulation and antifouling function of the panel, the structure that can increase the efficiency of solar energy, and many others must be considered [9].

With the advancement of solar panel technology, organic photovoltaic, high-efficiency, and high-performance panels have been developed [10,11,12,13,14]. However, conventional solar panels are usually black or blue, which is not preferred by most users. In buildings where aesthetics is important, front colored glass pieces that can withstand the harsh environmental conditions while transmitting light to the solar panel are required. To minimize the decrease in the efficiency of solar power generation while securing aesthetics, various studies on front colored glass have been reported [15,16,17,18,19]. Colored glass maximizes reflection at a desired wavelength and shows a variety of colors when viewed externally, thereby concealing the solar panel while ensuring aesthetics and limited reflection to a specific wavelength are promoted. Since it has high transmittance at other wavelengths, except for the reflected spectra, it minimizes the decrease in the efficiency of the solar panel.

Kromatix technology [19] applied a multilayer coating on the inner glass surface by evaporation in a low-pressure plasma process. Low-pressure plasma process such as atomic layer deposition has great potential to produce thin, uniform, and conformal films with accurate thickness while minimizing substrate damage. Unfortunately, most of the materials chosen for the multilayer structures have a low refractive index. As a result, thick layers to obtain bright and desired colors are required. This method is expensive, has a long deposition time, produces low transmittance layers, and is not applicable to commercial BIPV systems. Recently, we implemented colored glass for BIPV through multilayer deposition of alternating high and low refractive index metal oxides and metal nitrides [20,21,22,23]. Some of these oxide and nitride layers have been used in thin-film optical applications because of their high transmittance and stable optical properties [24]. However, for large area applications, deposition of high refractive index materials such as metal oxides and metal nitrides is sensitive to thickness, making it difficult to exhibit uniform properties in one film. In addition, the color changes easily depending on the viewing angle. Therefore, it is not suitable for implementing uniform, stable, and large area colored glass. Various studies are currently investigating a method of developing colored glass through a polymer-based solution process instead of vapor deposition [25]. This can resolve the problem of non-uniformity in large area applications and many other challenges that are mostly associated with the physical vapor deposition processes. Moreover, the marketability of most conventional colored glass is difficult due to the high material price and the need for a lamination process using ethylene vinyl acetate (EVA). Therefore, there is a need to develop economical colored glass that can secure the aesthetic elements of buildings while having high transmittance that can be applied to BIPV systems.

In this paper, a manufacturing process for colored glass is proposed by depositing pearlescent pigments using a solution process technique. Pearlescent pigments are composed of plate-shaped crystals unlike general pigments [26]. The reflected light is generated through the interference of light caused by the difference in the refractive index of the plate-shaped crystal and that of the pigments, resulting in appealing colors like natural pearls or metallic lusters. These are special pigments due to their optical effect [26]. Analysis of the optical properties of the color films are made through the measurement of transmittance and reflectance. In the lamination process of the manufactured front colored glass onto the solar panel, the process time and cost can be reduced by omitting the EVA film lamination used in the existing processes by rather leveraging on the adhesive properties of the NOA. The spin-coated color films are directly laminated on solar cells by UV curing in a one-step process. Finally, the effect of the colored glass on the solar power generation efficiency and stability of the BIPV module are analyzed. It was demonstrated that more than 94% of solar efficiency can be achieved by PV cells laminated with colored glass. With the development of this technology, it is expected that colored glass with free color choice will be possible in the future, and will be applied to low-cost BIPV systems with improved aesthetics and energy efficiency.

## 2. Materials and Methods

### 2.1. Fabrication of Colored Glass Using Pearlescent Pigments

**Figure 1:**
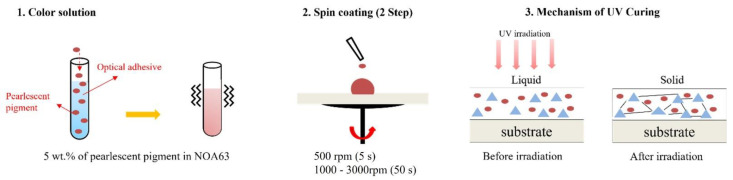
Solution formulation and colored glass fabrication process.

In this section, we present the manufacturing process of colored glass by pearlescent pigments in a solution process and propose a one-step UV curing to laminate the colored glass on solar cells. The pearlescent pigments used for preparing the color solutions are A-781K (Splendor Blue), AX-701K (Dazzling Gold), AX-741 (Dazzling Red), AX-761 (Dazzling Violet), AX-791K (Dazzling Green), and AX-901K (Dazzling Standard). All the pearlescent pigments were supplied by CQV (Jincheon, Korea). The particle size of the pearlescent pigments ranged from 5–41 μm and were uniformly mixed in NOA63 matrix (Norland Products, Jamesburg, NJ, USA) (Figure S1). The glass substrates (2.5 × 2.5 cm^2^) used in this study were mist type and normal type (NURI CORP, Seongnam, Korea). The mist glass creates a scattering effect on the back to show a distinct color in scattered light. The two types of glass substrates were cleaned with de-ionized water and acetone for 15 min each in an ultrasonic bath, boiled for 15 min with isopropyl alcohol (IPA), and dried in an oven at 150 °C for 15 min. The solution was prepared with 5 wt.% of pearlescent pigment in NOA63. A color layer was formed on a glass substrate by using the spin coating method with a color solution. The colored glass fabrication process is shown in Figure 1. The glass substrate was placed on a spin coater and the prepared color solution was applied. Spin coating was done at 500 rpm for 5 s to evenly disperse the applied solution. Next, the spin coating speed was increased between 1000 rpm and 3000 rpm for 50 s to tune the thickness of each color film. Lastly, the colored glass was cured in 15 min by an ultraviolet (UV) with a wavelength of 365 nm. The thickness of the color films was measured by alpha step measurement (Dektak 150, Veeko, Hong Kong) while the optical transmittance and reflectance were measured with an ultraviolet visible light near-infrared spectrophotometer (Lambda 950 UV-vis-NIR, PerkinElmer, Waltham, MA, USA). The incertitude of the transmittance and reflectance measurement was within 0.004%. Large area colored glass samples were coated with a slot-die coater (PBC-200, PEMS, Deajeon, Korea).

### 2.2. Lamination Process of Colored Glass on Solar Cells

**Figure 2:**
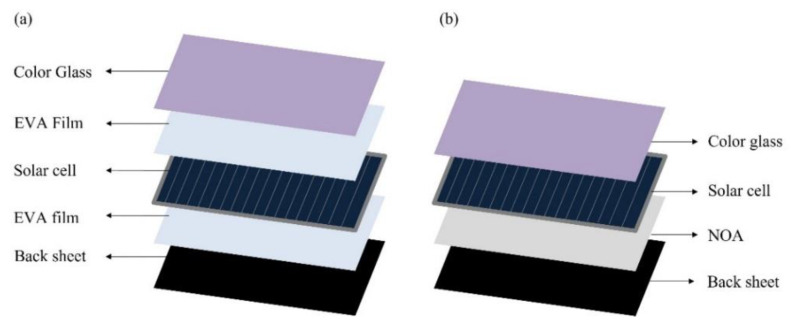
Solar cell lamination process for the BIPV system. (**a**) Existing process using EVA film; (**b**) proposed lamination process without EVA film.

After spin coating the color solution on the glass substrate, it was directly attached to the solar cell to perform the lamination process. The commercial PV cells were purchased from SHINSUNG E&G (Korea, Model: SH-2180S5P-D) and used as is. Figure 2 shows the components of the conventional and proposed solar cell with laminated colored glass, respectively. As shown in Figure 2a, the conventional system employs EVA as the laminating film. However, as shown in Figure 2b, the proposed NOA63-based color solution was spin coated on the glass substrate and directly laminated to the solar cell. In addition, NOA63 was coated on the back sheet and laminated at the rear side of the solar panel. In the proposed system, the entire structure is cured using UV with a power of 0.61 mW/cm^2^ at 365 nm. The use of EVA in the conventional lamination was eliminated completely, thereby reducing the process time and cost significantly. The colored glass for the solar cell test was fabricated on 9×4 cm^2^ normal glass at a spin coating speed of 1500 rpm, and the power generation efficiency was measured using a solar cell efficiency measuring device (McScience, K201LAB50, Suwon, Korea).

## 3. Results and Discussion

### 3.1. Thickness of Color Films and the Optical Characteristics of the Colored Glass

The thickness of the thin film prepared by spin coating was investigated. Table 1 shows the structure of the colored glass samples A, B, C, D, E, and F. Generally, each sample structure is of the order: glass/color film, where the reflectance range and color of each film is indicated in the third and fourth column, respectively.

Pearlescent pigments consist of substrates with a high aspect ratio in a layered structure. They are usually synthetic or natural micas that have been coated with tin oxide, titanium dioxide (TiO_2_), alumina, silicon dioxide or other metal oxides—laminar crystal. The laminar crystals can vary in size, shape, and thickness. The color of the pearlescent pigments depends on the type of substrate and the pigments used. Natural micas, synthetic micas, glass flakes, and alumina are examples of substrates used. The colors come from the interference effect from the laminar crystal [20,21,22,23]. The interference color is derived by coating TiO_2_ on these high aspect ratio substrates to a very specific thickness. Due to the difference in refractive indices between the oxide and substrate layers, the incident wavelength of white light striking the coated flake is split into very specific reflected and transmitted components. For example, a thin layer of TiO_2_ results in a white pearlescent effect, while a thick layer of TiO_2_ results in a corresponding red transmissive component or a green reflective component with a rainbow effect. Any color in the spectrum is possible by fine-tuning the thickness of TiO_2_. As light continues to pass through the film, it reflects more from the surrounding flakes in a similar way, resulting in a series of colors. In a similar way, when the thickness of single-layer iron oxide (Fe_2_O_3_) or one combined with TiO_2_ is changed, a non-metallic effect with a pearlescent luster is created. However, due to the absorption color component of Fe_2_O_3_, the observer perceives a metallic luster instead. A thin layer of Fe_2_O_3_ takes on a gold color and as it gets thicker it goes through bronze, copper, and maroon color effects. In addition, various colors can be implemented by using various colorants. In the colored glass samples A, B, C, D, E, and F, A-781K, AX-701K, AX-741, AX-761, AX-791K, and AX-901K pearlescent pigments are used, respectively. These commercial pearlescent pigments are among several others that can be freely selected to fabricate colored glass through our proposed manufacturing process. It is demonstrated that the required optical characteristics such as reflectance, transmittance, and morphology can be determined by the particle size distribution, pigment color, pigment substrate, and coating parameters of the manufacturing process.

**Figure 3:**
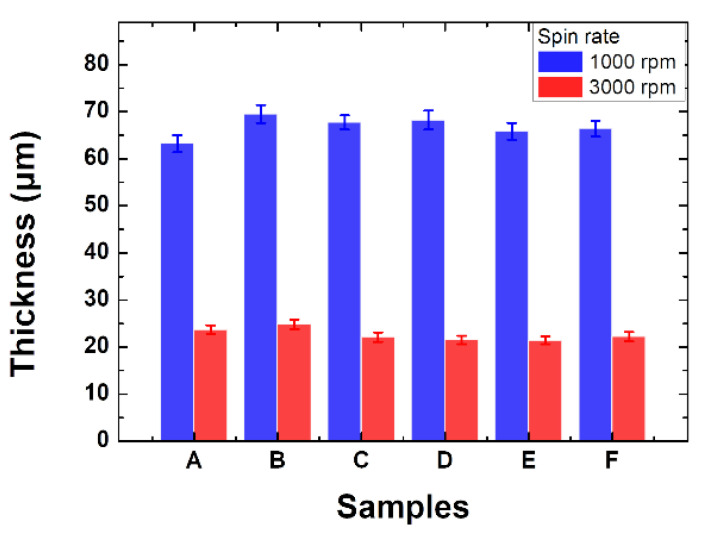
The thickness of the thin film formed according to the spin coating speed of 1000 rpm (solid blue), and 3000 rpm (solid red), respectively.

Figure 3 shows the thickness of the films deposited at 1000 rpm and 3000 rpm, respectively. Samples spin-coated at 1000 rpm and 3000 rpm resulted in color layers with thickness in the range of 63–70 μm and 21–23 μm, respectively. Thus, it is demonstrated that thin films of 21–70 μm can be deposited by simply adjusting the spin coating speed in the range of 1000 to 3000 rpm. The thin film thickness is an important factor controlling the transmittance and the intensity of the reflectance of the colored glass. This is because the distribution of pearlescent pigment varies according to the spin coating speed and the thickness of the thin film, leading to control of the optical transmittance of the colored glass.

**Figure 4:**
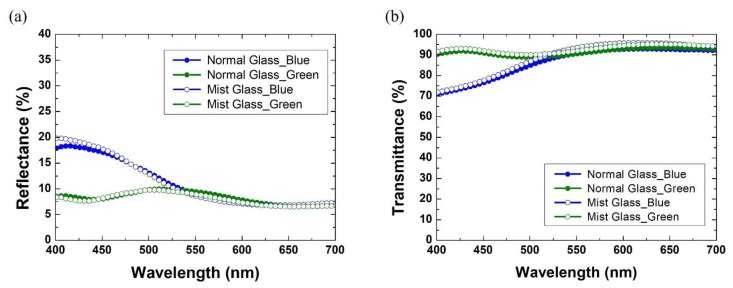
(**a**) Reflectance and (**b**) transmittance spectra of color films spin coated at 1000 rpm on normal and mist glass substrates, respectively. Blue color solutions were deposited on normal glass (blue, solid circle) and mist glass (blue, open circle). Green color solutions were deposited on normal glass (green, solid circle) and mist glass (green, open circle).

The optical characteristics of the colored glass was investigated. First, the effect of the glass substrates on the reflectance and transmittance spectra is analyzed. Figure 4 shows the reflectance and transmittance spectra of blue and green color films spin coated at 1000 rpm on normal and mist glass substrates, respectively. The mist glass has the same thickness and refractive index of 1.52 as the normal glass. However, it causes a scattering effect with little effect on the transmittance and reflectance spectra. The optical properties are determined by the optical path difference (*OPD*) [27]:

(1) OPD=2×n×d×cosθ

where θ is the angle of incidence, n is the refractive index, and d is the thickness of the color film. For the same angles of incidence, the resultant *OPD* in both glass types are the same, yielding similar optical properties. Thus, it is demonstrated that similar transmittance, and color brightness are achieved in both glass types, regardless of the color of the pearlescent pigment. However, due to the scattering effect, mist glass with the color film effectively hides the solar panel while maintaining efficiency and color brightness.

**Figure 5:**
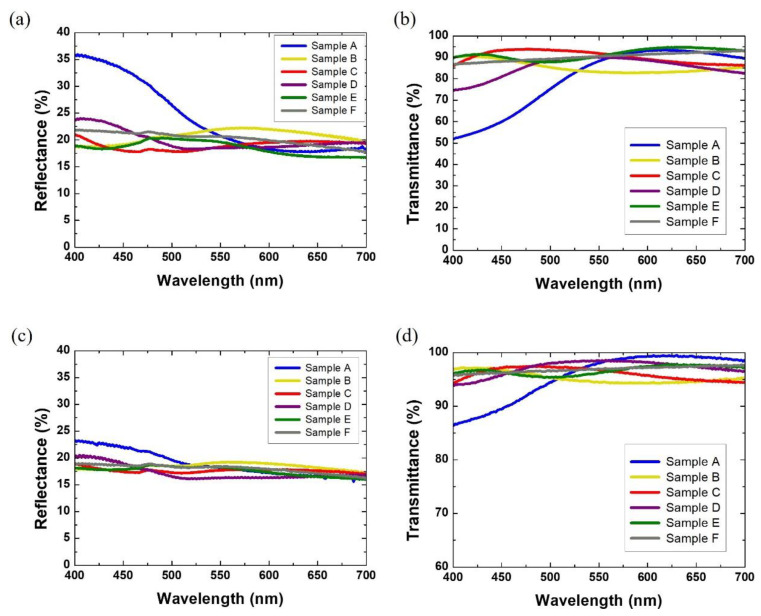
(**a**) Reflectance and (**b**) transmittance spectra of colored glass samples produced at a spin coating speed of 1000 rpm. (**c**) Reflectance and (**d**) transmittance spectra of colored glass samples produced at a spin coating speed of 3000 rpm: A (blue solid line), B (yellow solid line), C (red solid line), D (violet solid line), E (green solid line), F (grey solid line).

Next, the effect of thickness (spin coating speed) of the color films on the optical characteristics of the colored glass was investigated. Figure 5a,b shows the reflectance and transmittance spectra of colored glass samples A to F prepared at a spin coating speed of 1000 rpm. The reflectance spectra in the visible range of samples A, B, C, D, E, and F, fabricated at a spin coating speed of 1000 rpm, are shown in Figure 5a. Samples A and D achieved a maximum reflectance of about 35% and 24% for the blue and violet wavelength regions, respectively; thus, the samples exhibit blue and violet colors, respectively. Sample B shows a maximum reflectance of 25% near the wavelength range of 450–570 nm. Sample C exhibits a maximum reflectance of 21% at 700 nm and about 19% at 400 nm, resulting in a pale red color. Sample E exhibits evenly reflected spectra with a maximum reflectance of 21% in the wavelength range of 490–550 nm, yielding a green colored glass. Sample F exhibits even reflectance distribution over the entire visible wavelength spectrum; hence, a gray colored glass is produced. Figure 5b shows the transmittance spectrum of the samples prepared at a spin coating speed of 1000 rpm. All samples show maximum transmittance of 85% or more for light whose wavelengths are not within the maximum reflectance range.

The reflectance and transmittance spectra of samples prepared at 3000 rpm spin coating speed are shown in Figure 5c,d, respectively. Generally, it can be noticed that the samples made at a spin coating speed of 3000 rpm have similar characteristics as samples made at a spin coating speed of 1000 rpm. However, the maximum reflectance decreased, and the maximum transmittance increased. Even though the same color is achieved but with low reflectance, the solar efficiency can be increased. The results in Figure 5 are verified by optical microscopy images (Figure S2). As the spin coating speed increases, the distribution of pearlescent pigment decreases. This results in increased transmittance and decreased reflectance, respectively. Conversely, at low spin coating speeds, the intensity of reflectance decreases and the transmittance increases. This phenomenon may also be related to the thickness of the thin film being formed. As the film becomes thicker, the active area where the pearlescent pigment can be distributed increases. Thus, the maximum transmittance and reflectance can be adjusted according to the distribution of the pearlescent pigments.

**Figure 6:**
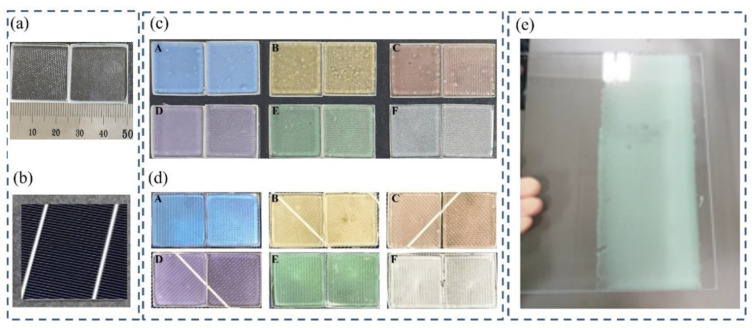
Actual photographs of (**a**) Normal and mist glass without a color layer, (**b**) PV cell, (**c**) samples A to F made at a spin coating speed of 1000 rpm, (**d**) laminated samples A to F (Left: Normal glass, right: mist glass), and (**e**) slot-die coated colored glass.

The photographs of the reference and fabricated samples are shown in Figure 6. The reference (bare) mist glass and the PV cell are shown in Figure 6a,b, respectively. As it is for conventional solar panels, the commercial PV cell shows a black color. Figure 6c shows the actual photographs of the colored glass pieces on a black background. It is demonstrated that uniform films are formed on the substrate without additional surface treatment. In the conventional solar module, the lamination process using EVA film is involved. The lamination process using the EVA film has disadvantages of long process time and high temperature curing requirements that are not compatible with commercial flexible applications. However, by using an NOA matrix, it is possible to laminate the colored glass in the BIPV system by adopting a solution process in a short time and at a low temperature without additional processes and materials. The color solution is spin coated on the substrate and a UV curing process conducted to laminate all elements of the module. Figure 6d shows the actual photographs of solar cells with the colored glass pieces after the lamination process. Generally, each sample structure is of the order: glass/color film/PV cell. Implementing a solution process technique to fabricate colored glass provides the best route in achieving largescale commercial applications. Beyond lab-scale spin coating, we demonstrated that the color solutions can be applied using slot-die coating. Figure 6e shows a slot-die coated colored glass. It is demonstrated that uniform colored glass can be achieved using large-scale printing and roll-to-roll technologies, such as slot-die coating.

It was demonstrated that the optical characteristics of the colored glass were not changed by the lamination process. The investigation has shown that colored glass produced by spin coating pearlescent pigments mixed in NOA matrix can effectively meet both aesthetic and solar efficiency requirements. This technique also offers the advantage of a one-step lamination process. Therefore, it is expected to be applied to the market to develop front colored glass for BIPV systems and manufacture cost-effective solar modules.

### 3.2. Solar Efficiency of PV Cells with Colored Glass and Stability Analysis of the Colored Glass

Large area colored glass for solar efficiency measurement and stability test was fabricated on 9×4 cm^2^ normal glass at a spin coating speed of 1500 rpm. The transmittance of these samples exhibits intermediary levels compared to the transmittance spectra of samples prepared by spin coating at 1000 rpm and 3000 rpm, respectively (Figure S3). The colored glass samples were laminated to the solar cell by a one-step lamination, as previously proposed.

**Figure 7:**
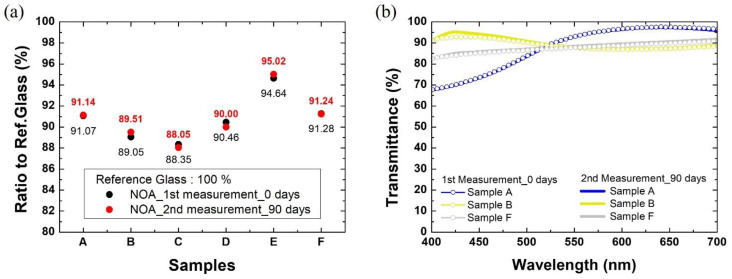
Characteristics and stability evaluation results of BIPV modules manufactured by colored glass A–F (**a**) power generation efficiency, (**b**) transmittance.

Figure 7a shows the ratio of the solar efficiency of the PV module laminated with bare reference glass to PV modules laminated with the colored glass samples A–F, respectively. The current–voltage characteristics and performance metrics of the PV modules with reference glass are shown in Figure S4 and Table S1, respectively. Samples A and B show maximum transmittance of 97% and 95% at 641 and 425 nm, respectively, and power generation efficiency of 91.07% and 89.05%, compared to the reference glass. Samples C and D show the maximum transmittance of 89% and 96% at 485 and 543 nm, respectively, and the power generation efficiency of 88.35% and 90.46%, compared to the reference glass, respectively. Samples E and F showed the maximum transmittance of 94% and 92% at 440 and 700 nm, respectively, and the power generation efficiency of 94.64% and 91.28%, compared to the reference glass, respectively. It is noticed that sample A, which has the highest maximum transmittance, is not as efficient as sample E or F. Sample A has the maximum transmittance, but only in a certain wavelength range (570–700 nm). Compared to sample A, the maximum transmittance of Sample E is lower by about 3% but covers a wide wavelength range (400–470 nm, and 570–700 nm) and shows the highest power generation efficiency. Therefore, to increase the solar power generation efficiency, it is desirable to maintain high transmittance in a wide wavelength range; this is also true for sample F. In addition, the photovoltaic power spectrum of a typical solar cell is made in a wide wavelength range, including ultraviolet and infrared rays from 200 to 2500 nm. Since our study is focused on the design of colored glass only for the visible wavelength range, the effect of the wavelength ranges in the ultraviolet and the infrared ranges on the solar efficiency has not yet been covered. However, the ultraviolet-visible infrared spectrum can clearly affect the photovoltaic power generation.

The BIPV module must ensure the stability of the colored film in external environmental conditions. Figure 7a,b show that the power generation efficiency and transmittance of colored glass were maintained for three months under ambient conditions, respectively, without significant degradation. It was confirmed that the degree of change in power generation efficiency according to the test period is within 0.5%. This is within the margin of error. In addition, it is confirmed that the change in optical properties is insignificant. This shows the stability of the colored glass made by the NOA matrix—the stability of the color films made by the colored glass produced by spin coating the pearlescent pigment mixed in the NOA matrix can effectively meet both aesthetic and solar efficiency requirements. Therefore, it is expected to be applied to the development of full-colored glass for BIPV systems and cost-effective solar module manufacturing.

## 4. Conclusions

In this paper, we proposed a solution process to fabricate front colored glass for BIPV systems using a color mixture composed of pearlescent pigments and NOA63 matrix. Compared to the conventional evaporation techniques, our approach offers a simple and a cost-effective process to achieve remarkable aesthetic functional modules. The optical characteristics and thickness of the color films can be easily controlled by adjusting the spin coating speed. The fabricated colored glass achieved a high transmittance of 85% or more in the wavelength range not optimally reflected. In addition, a simple and inexpensive lamination process of the colored glass on the solar panel was introduced by omitting the EVA film. The one-step lamination was achieved by conducting a UV curing of the spin-coated color films directly on the solar cell. It was demonstrated that the PV with the colored glass could achieve a high solar efficiency of up to 95%, with an insignificant change in the color film over time. The proposed module is suitable for implementing large area colored glass for BIPV systems by adopting printing technologies. Through the development of this technology, colored glass with free color selection is expected in the future and will be applied to low-cost BIPV systems with improved aesthetics.

## Figures and Tables

**Table 1:** Structure of the fabricated colored glass.

Sample	Layer Structure	Reflectance Range (nm)	Color
A	Glass/A-781K + NOA63	460–480	Splendor Blue
B	Glass/AX-701K + NOA63	590–610	Dazzling Gold
C	Glass/AX-741K + NOA63	630–650	Dazzling Red
D	Glass/AX-761K + NOA63	410–430	Dazzling Violet
E	Glass/AX-791K + NOA63	520–540	Dazzling Green
F	Glass/AX-901K + NOA63	350–780	Dazzling Standard

## Data Availability

The data presented in this study are available on request from the corresponding author.

## Supplementary Materials

The following supporting information can be downloaded at: https://www.mdpi.com/article/10.3390/ma15072627/s1, Figure S1: Color solutions composed of pearlescent pigments and optical adhesives (NOA) matrix. From left, A-781K (Splendor Blue), AX-701K (Dazzling Gold), AX-741 (Dazzling Red), AX-761 (Dazzling Violet), AX-791K (Dazzling Green), and AX-901K (Dazzling Standard), Figure S2: Optical microscopy image of colored glass samples produced at a spin coating speed of (a) 1000 rpm, (b) 2000 rpm, (c) 3000 rpm. Figure S3: transmittance spectra of colored glass samples produced at a spin coating speed of 1500 rpm, Figure S4: Electrical characteristics of the BIPV module with reference glass (a) voltage (X-axis), current (left Y-axis), power (right Y-axis), (b) voltage (X-axis), current density (left Y-axis), Table S1: Performance metrics of BIPV module with reference glass.
