# Supplementary material for: Fabrication of Color Glass with High Light Transmittance by Pearlescent Pigments and Optical Adhesive
Source: Materials (Basel). 2022 Apr 2;15(7):2627. doi: 10.3390/ma15072627 (PMC9000421; doi:10.3390/ma15072627)
Supplement: Supplementary file 1 [file materials-15-02627-s001.zip › materials-1606833-supplementary.pdf]

materials

Supplementary Materials

Fabrication of color glass with high light transmittance by pearlescent pigment and optical adhesive

Hyeon-Sik Ahn[1], Akpeko Gasonoo[2], Seong-Min Lim[1], Jae-Hyun Lee[3], and Yoonseuk Choi[1,*]

[1] Department of Electronic Engineering, Hanbat National University, Daejeon 34158, Republic of Korea; princass123@naver.com (H.-S.A.); seongmin625@naver.com (S.-M.L.)
[2] Research Institute of Printed Electronics & 3D Printing, Hanbat National University, Daejeon 34158, Republic of Korea; amoebatheo2009@gmail.com
[3] Department of Creative Convergence Engineering, Hanbat National University, Daejeon 34158, Republic of Korea; jhyunlee@hanbat.ac.kr
* Correspondence: ychoi@hanbat.ac.kr; +82-42-821-1134

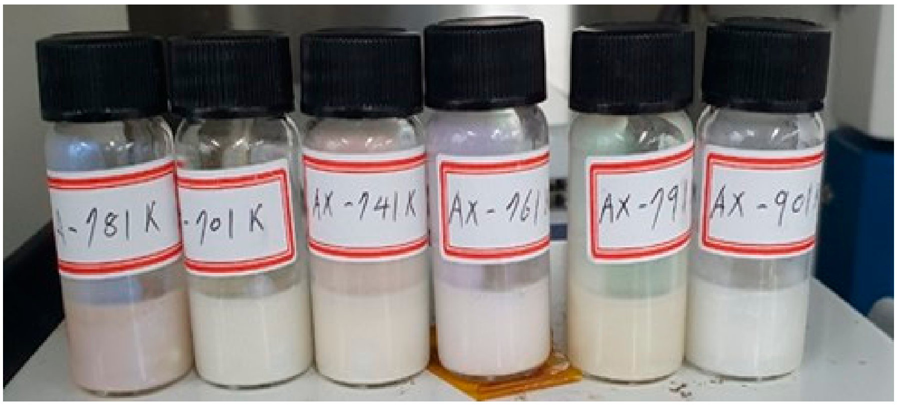

Figure S1. Color solutions composed of pearlescent pigments and optical adhesives (NOA) matrix. From left, A-781K (Splendor Blue), AX-701K (Dazzling Gold), AX-741 (Dazzling Red), AX-761 (Dazzling Violet), AX-791K (Dazzling Green) and AX-901K (Dazzling Standard

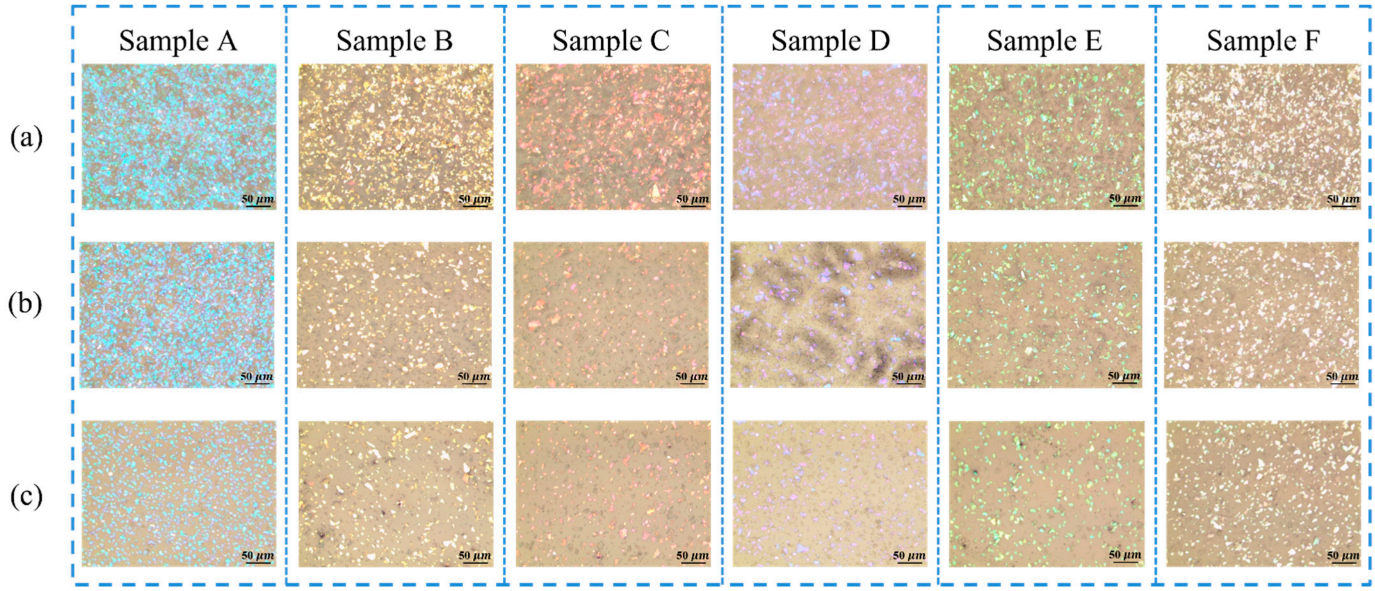

Figure S2. Optical microscopy image of color glass samples produced at a spin coating speed of (a) 1000 rpm, (b) 2000 rpm, (c) 3000 rpm.

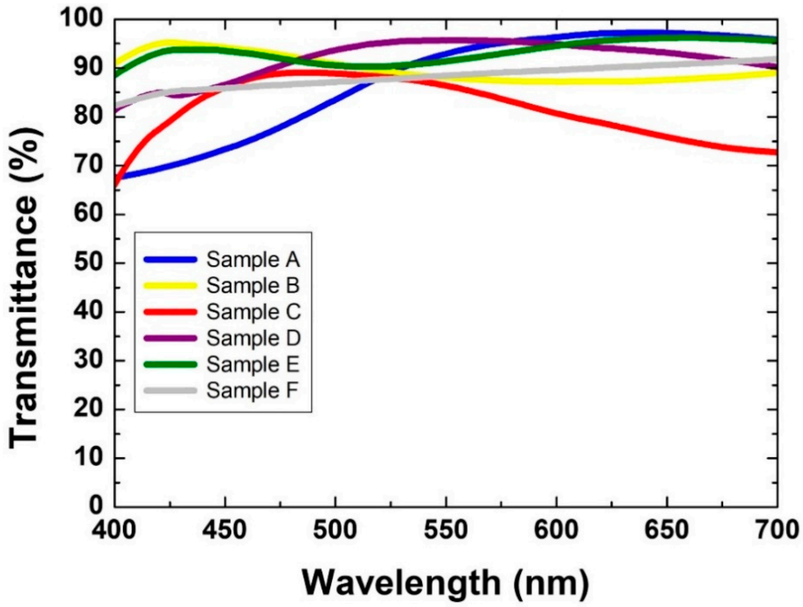

Figure S3. Transmittance spectra of color glass samples produced at a spin coating speed of 1500 rpm.

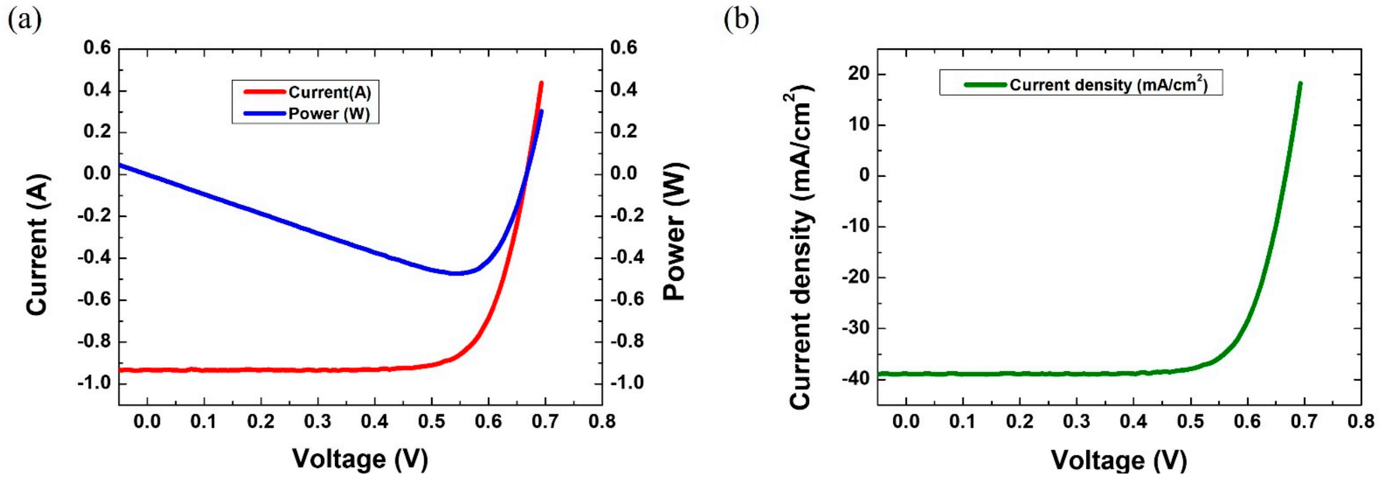

Figure S4. Electrical characteristics of the BIPV module with reference glass (a) voltage (x-axis), current (left Y-axis), power (right Y-axis), (b) voltage (x-axis), current density (left Y-axis)

Table S1. Performance metrics of BIPV module with reference glass

V_{oc} (V)	0.667
I_{sc} (A)	0.933
J_{sc} (mA/cm2)	38.85
P_{max} (W)	0.473
V_{max} (A)	0.541
I_{max} (A)	0.874
Efficiency (%)	19.70
